# Supplementary material for: Co-Benefits of Energy Structure Transformation and Pollution Control for Air Quality and Public Health until 2050 in Guangdong, China
Source: Int J Environ Res Public Health. 2022 Nov 14;19(22):14965. doi: 10.3390/ijerph192214965 (PMC9690161; doi:10.3390/ijerph192214965)
Supplement: Supplementary file 1 [file ijerph-19-14965-s001.zip › ijerph-1934677-supplementary.pdf]

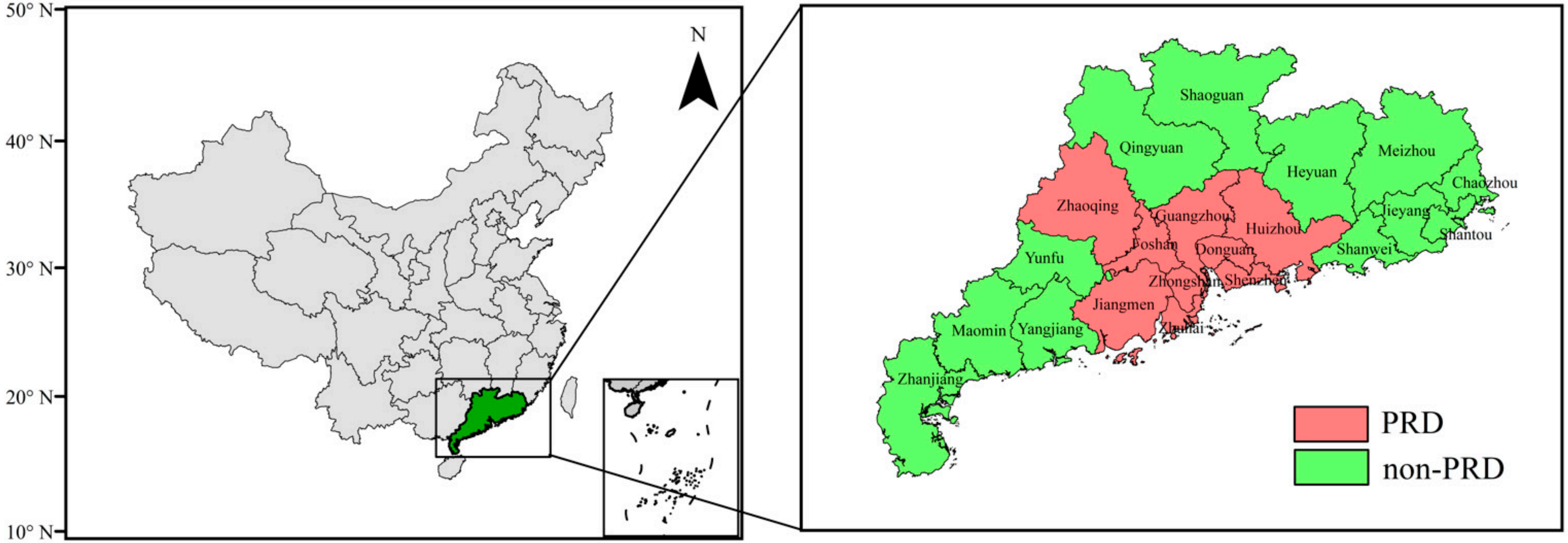

Figure S1. The location and regional division of Guangdong Province.

Table S1. The key parameters and assumptions in the IPAC model for the future social development of Guangdong.

Category	Sub-Category	Units	2015	2020	2025	2030	2035	2040	2045	2050
GDP growth rate		%	6.8	6.1	5.4	4.3	3.6	3.1	2.7	2.3
Industrial structure	Primary industry	%	4.6	4.3	4.1	4.0	3.8	3.7	3.5	3.4
	Secondary industry	%	44.8	40.0	37.3	34.0	32.4	30.1	29.3	27.0
	Tertiary industry	%	50.6	55.7	58.7	62.0	63.8	66.2	67.2	69.6
Population		10000 people	10849	11283	11452	11621	11621	11621	11215	10808
Urban rate		%	69	75	77	79	80	80	81	82
Urban population		10000 people	7485.81	8462.25	8821.34	9180.59	9239.26	9296.8	9080.19	8862.56
Population per urban household		people	2.95	2.87	2.82	2.7	2.65	2.6	2.54	2.5
Number of urban households		10000 household	2537.563	2948.519	3174.816	3400.219	3488.211	3575.692	3560.366	3545.024
Urban residential space per capita		Square meters	32.1	39	41	43	44	45	45	45

Number of household appliances per 100 urban households	Refrigerator		75.3	97	99	100	100	100	100	100
	Washing machine		71	95	98	100	100	100	100	100
	Air conditioner		144	220	240	260	260	260	260	260
	Water heater		84	105	105	105	105	105	105	105
	Microwave oven		39	48	64	80	88	95	95	95
	Television		100	110	135	160	165	170	170	170
Household appliance use time in urban areas	Washing machine	Minutes/week	110	130	155	180	180	180	180	180
	Air conditioner	Hours/year	1100	1300	1500	1700	1750	1800	1850	1900
	Water heater	Minutes/week	245	269.5	310.4	350.35	359.5	367.8675	377.6	386.3
	Microwave oven	Minute/week	40	52	60	68	69	70	70	70
	Television	Hours per TV/week	21	26	26	26	26	25	25	25
Rural population		10000 people	3363.19	2820.75	2631.83	2440.41	2382.26	2324.2	2135.12	1945.44
Population per rural household		people	4.2	4.1	4.0	3.9	3.8	3.7	3.6	3.5
Number of rural households		10000 household	800.7595	687.9878	657.1298	625.7462	627.3761	628.1622	592.5512	555.8445
Rural		Square	42.1	50	55	59	63	67	69	70

residential space per capita		meters								
Number of household appliances per 100 rural households	Refrigerator		78	99	100	100	100	100	100	100
	Washing machine		65	92	96	100	100	100	100	100
	Air conditioner		62	150	180	210	215	220	220	220
	Water heater		77	99	100	100	100	100	100	100
	Microwave oven		21	39	60	80	88	95	95	95
	Television		117	120	133	145	148	150	150	150
Household appliance use time in rural areas	Washing machine	Minutes/week	105	125	153	180	180	180	180	180
	Air conditioner	Hours/year	950	1100	1400	1700	1750	1800	1850	1900
	Water heater	Minutes/week	217	247	285	322	330	338	347	355
	Microwave oven	Minute/week	40	52	60	68	69	70	70	70
	Television	Hours per TV/week	22	29	30	30	30	29	29	28
Building areas	Residential buildings	10000 square meters	381885	471065	504908	538750	556414	574077	554537	534996
	Service buildings		122203	150741	161571	172400	178053	183705	177452	171199
	Shopping mall buildings		8400	11004	12820	14635	16684	18733	20794	22855

	Restaurant buildings		235	286	321	355	391	426	452	477
	Office buildings		54991	67833	72707	77580	80124	82667	79853	77039
Number of vehicles		10000 vehicles	1689	2560	3341	4121	4575	5028	5103	5178
Number of passenger vehicles		10000 vehicles	991	1502	2171	2839	3293	3747	3785	3822
Air transport turnover		100 million passenger-kilometer	801	1397	1950	2502	3289	4075	4776	5477
Water transport turnover		100 million tonne-kilometer	19845	24500	28713	32926	36532	40137	42237	44336
Production of major industrial products	Crude steel	10000 tons	1762	3229	2257	1284	1137	989	890	791
	Cement		14530	16712	15208	13704	11306	8907	7316	5725
	Flat glass		9146	9998	9948	9898	9651	9403	8980	8557
	Vinyl		248	348	385	421	409	396	384	372
	Paper and cardboard		2178	2223	2457	2690	2610	2529	2453	2377
	Aluminum products		538	470	473	475	452	428	407	385

Table S2. WRF-Chem model configuration.

Process	WRF–Chem Option
Microphysics	Lin
Long-wave radiation	RRTM
Short-wave radiation	Goddard
Surface layer	Monin-Obukhov (Janjic Eta) scheme
Land surface model	Noah
Urban canopy model	Single-layer, UCM
Boundary layer scheme	YSU
Cumulus parameterization	New Grell (G3)
Photolysis scheme	Fast-J
Gas-phase mechanism	SAPRC99
Aerosol mechanism	MOSAIC

The simulation results for $PM_{2.5}$ and MDA8 from the WRF-Chem model were evaluated by comparing with the monitoring data released by the China National Environmental Monitoring Center. A total of 101 national air quality monitoring stations in Guangdong were used to evaluate the model performance, and the simulated concentration of grids where the 101 stations are located was chosen to make the comparison. As can be seen in Figure S2, the model fits well for the concentration of $PM_{2.5}$ and MDA8 overall; the error between the simulated and corresponding monitoring values at all

stations is less than one, with a small root mean square error on average.

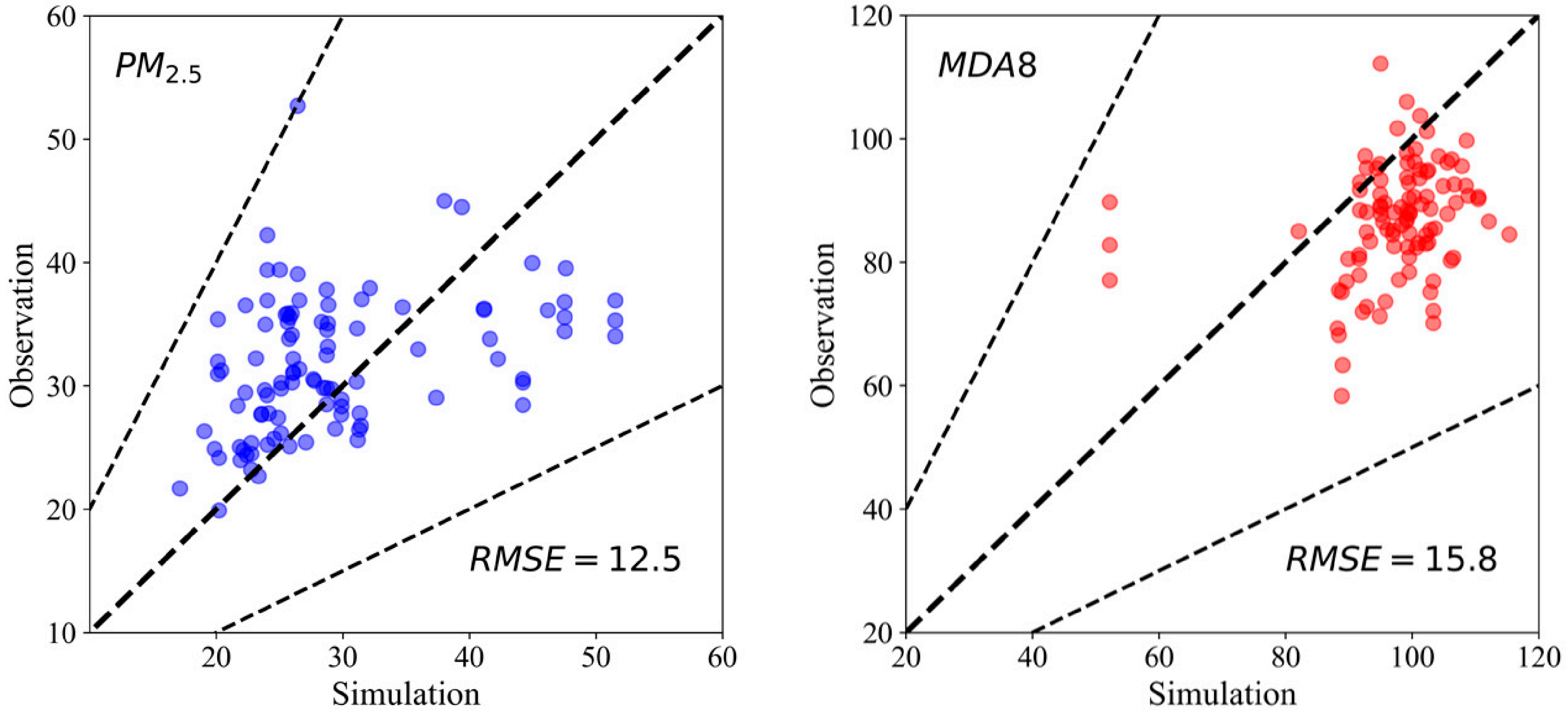

Figure S2. Validation of model performance, unit: μg/m^3. The heavy dash line indicates the 1:1 line, while the lighter dashed lines indicate the 1:2 and 2:1 lines.
